# Association between adverse childhood experiences, bullying, self-esteem, resilience, social support, caries and oral hygiene in children and adolescents in sub-urban Nigeria

**DOI:** 10.1186/s12903-020-01160-0

**Published:** 2020-07-11

**Authors:** Morenike Oluwatoyin Folayan, Olakunle Oginni, Olaniyi Arowolo, Maha El Tantawi

**Affiliations:** 1 Obafemi Awolowo University Teaching Hospitals Complex, Ile-Ife, Nigeria; 2 Department of Child Dental Health, Obafemi Awolowo University, Ile-Ife, Nigeria; 3 Faculty of Dentistry, Alexandria University, Qism Bab Sharqi, Egypt

**Keywords:** Adverse childhood events, Nigeria, Children, Adolescents, Psychosocial factors, Culture

## Abstract

**Background:**

Adverse childhood experiences (ACE) and bullying have negative effects on oral health. Promotive assets (resilience, self-esteem) and resources (perceived social support) can ameliorate their negative impact. The aim of this study was to determine the association between oral diseases (caries, caries complications and poor oral hygiene), ACE and bully victimization and the effect of access to promotive assets and resources on oral diseases.

**Methods:**

This was a secondary analysis of data collected through a cross-sectional school survey of children 6–16-years-old in Ile-Ife, Nigeria from October to December 2019. The outcome variables were caries, measured with the dmft/DMFT index; caries complications measured with the pufa/PUFA index; and poor oral hygiene measured with the oral hygiene index-simplified. The explanatory variables were ACE, bully victimization, resilience, self-esteem, and social support. Confounders were age, sex, and socioeconomic status. Association between the explanatory and outcome variables was determined with logistic regression.

**Results:**

Of the 1001 pupils with complete data, 81 (8.1%) had poor oral hygiene, 59 (5.9%) had caries and 6 (10.2%) of those with caries had complications. Also, 679 (67.8%) pupils had one or more ACE and 619 (62.1%) pupils had been bullied one or more times. The median (interquartile range [IQR]) for ACE was 1(3), for bully victimization was 1(5), and for self-esteem and social support scores were 22(5) and 64(34) respectively. The mean (standard deviation) score for resilience was 31(9). The two factors that were significantly associated with the presence of caries were self-esteem (AOR: 0.91; 95% CI: 0.85–0.98; *p* = 0.02) and social support (AOR: 0.98; 95% CI: 0.97–1,00; *p* = 0.02). No psychosocial factor was significantly associated with caries complications. Self-esteem was associated with poor oral hygiene (AOR: 1.09; 95% CI: 1.09–1.17; *p* = 0.03).

**Conclusion:**

There was a complex relationship between ACE, bully victimization, access to promotive assets and resources by children and adolescents, and oral health. ACE and bully victimization were not associated with oral health problems. Though self-esteem was associated with caries and poor oral hygiene, the relationships were inverse. Promotive assets and resources were not associated with caries complications though resources were associated with lower prevalence of caries.

## Background

Adverse childhood experiences (ACE) are negative life events that are detrimental to brain development, and are root causes of diseases and mortality [1]. ACE occur before the age of 18 years [1], and include physical, sexual and emotional abuse and neglect, as well as bullying victimization and parental separation. They directly increase the likelihood of a child having poor oral health, including dental caries [2–4], and are also associated with adult health risks, such as cardiovascular diseases [5], diabetes mellitus [6] and smoking [7] which in turn, are associated with poor oral health in adulthood [8–10]. ACE further have negative impacts on the neuroendocrine-immune system and host defense mechanism [11] thereby causing high psychological stress [12] that increase the risk for poor oral hygiene and, thereby, caries [13].

Vasiliou et al. [14] developed a conceptual framework from the works of Shankardass [15] and Pearlin et al. [16], to explain the possible link between psychological stress and poor oral health. They suggested that ACE may translate into chronic stress in the absence of promotive assets (resilience, self-esteem) and resources (perceived social support). Chronic stress results in allostatic load, a cumulative physiological impact of chronic stress, that impacts negatively on oral health directly (by increasing inflammatory response which causes periodontal disease [17, 18]) or indirectly (by causing the adoption of unhealthy oral habits such as poor oral maintenance that can result in poor oral hygiene and caries).

Another adverse factor that may have a negative impact on oral health is bully victimization. Bully victimization is defined as the experience of distress or feeling of being controlled by others through aggression and/or power [19]. Good oral health enables children to have a healthy smile, which may be associated with less bully victimization [20]. Multiple studies indicate that poor dental aesthetics [21–23] and untreated caries [24] increase the risk for bully victimization. Poor oral hygiene causes halitosis, and halitosis is a risk factor for bully victimization [25]. In addition, poor dental aesthetics [26] and caries [27–29] have negative emotional and social effects on oral health-related quality of life because of their negative impact on self-esteem and self-concept. The history, frequency and consequences of bully victimization also affects oral health related quality of life [30, 31].

Protective factors can mitigate the relationship between ACE and bully victimization and children’s oral health [4, 32] by enhancing one’s ability to use available resources to manage life’s difficulties and maintain one’s health [33, 34]. One of these protective factors is high self-esteem [35]. Improving self-esteem was found to be effective for the management of children with cleft lip and palate [36]. Other individual factors that protect against the negative impact of adverse life events are one’s resilience [37] and access to support [38]. Resilience - an individual’s trait-like ability to demonstrate stable level of functioning despite adversity – acts as a buffer that facilitates positive outcomes despite exposure to adverse life events [39]. Social support also acts as a buffer against the stress that results from adverse life events by enhancing cognitive and emotional processing of the stressful event in a manner that is psychologically adaptive [40].

There is a scarcity of studies determining the relationship between children’s adverse experiences, bullying victimization, protective psychosocial factors and oral health for African populations, despite the identified relationship between these factors [41] and the importance of culture and context in moderating these relationships [42–44]. Individuals pull on external and internal resources to help them manage, cope with, or resolve tensions in health-promoting ways that reduce stress [33, 34]. Culture influences how individuals deal with tensions and how they identify resources to manage stresses [34]. Africans may be more collectivist-oriented than populations in the Global North who may value individualism and liberalism to a greater extent [45, 46].

This study was conducted in a suburban community in Nigeria, West Africa. This is a collectivist community where the rights and interests of individuals are subordinate to the good of the community. It is a community that holds traditional patriarchal beliefs and strong religious and extended family ties [47]. The study determined the relationship between ACE and exposure to bully victimization and oral health status accounting for factors that help overcome the negative effects of these risk factors. Specifically, the study explored the association between oral diseases (caries, caries complications and poor oral hygiene), ACE, bully victimization and promotive assets (resilience, self-esteem) and resources (perceived social support) [48] in schoolchildren in Ile-Ife, Nigeria.

## Methods

### Ethical consideration

Approval for the study was obtained from the Research and Ethics Committee of the Obafemi Awolowo University Teaching Hospitals Complex, Ile-Ife, Nigeria (ERC/2018/08/06). Permission was obtained from the Local Government Education Authority, Osun State, and the authorities of schools involved in this study.

Informed consent for study participation was obtained from the parents of all eligible pupils enrolled in the study and assent was obtained from children 12–16 years old. The consent and assent forms were sent to parents ahead of the school-visit date. On the day of the visit, only children who had the filled and signed informed consent forms and, where appropriate, the assent forms were included in the study. When parents/guardians had not signed an informed consent form but the child was keen to participate in the study, the child’s parent(s)/guardian(s) were called by telephone to seek verbal consent, and a filled written consent was obtained retroactively. The phone conversation was recorded. If the parent/guardian showed no interest in child/ward participation, the child was excluded from the study. Data were collected anonymously. Students did not receive reimbursement for study participation.

### Study design and study population

This is a secondary analysis of data collected to determine the association between caries and nutritional status. The primary study was a cross-sectional study that recruited children aged 6 to 16 years attending private and public primary and secondary schools in Ife Central Local Government Area, Ile-Ife, Osun State, Nigeria from October to December 2019. Children and adolescents with special health-care needs, those who were ill, and those who had fasted within a period of 3 months before data collection, were excluded from the study. The age 6-years was chosen as the lower limit because they would have developed the cognitive ability to respond to the questionnaire [49, 50].

### Sample size

The sample size for the primary study was determined according to the formula of Metcalfe [51] and using a caries prevalence of 13.9% as had been determined in a prior study in the population [52]. To recruit 168 children with dental caries, underweight, normal weight, overweight and obesity, 1209 children were required to give a power of 80%. The sample for the primary study was 1502.

### Sampling procedure

A multi-stage cluster sampling technique was used to recruit participants for the primary study. Children 6–10 years of age were recruited from primary schools, while those who were 11–16 years old were recruited from secondary schools.

First, schools were stratified into primary and secondary schools. The ratio of primary to secondary schools in the study population was 2:1 and the ratio of public to private school was 1:4. Next, 20 primary schools (3 public, and 17 private) and 10 secondary schools (2 public and 8 private) were randomly selected. At the schools, the class registration list was used to identify classes with the highest number of children. Children from the selected classes were asked to pick ballot papers with ‘yes’ or ‘no’ options. Those who picked ‘yes’ were recruited for the study.

### Data collection instruments

An interviewer-administered questionnaire collected data on participant’s sex, age at last birthday (6–11-year-old and 12–16-year-old), and child’s socioeconomic status [53]. Other sections of the questionnaire are as follows:

#### Adverse childhood experiences

were measured according to the 10-item Adverse Childhood Experiences Questionnaire, which provides a measure of cumulative life stress experienced during childhood [54]. These include experiences of parental verbal or physical assault, parental divorce, witnessing of maternal or grandmother’s physical abuse, experiences of emotional deprivation, sexual assault, and/or having a family member who is an alcoholic, mentally ill or an ex-convict. The instrument has been validated for use in Nigeria [55]. The response to each of the 10 questions is either ‘yes’ or ‘no,’ with possible score ranges from 0 to 10. The higher the score the more life adversities the child has faced.

#### Childhood bully victimization

was assessed with the victim subscale of the Illinois Bully Scale [56] and has been validated for use in Nigeria with a Cronbach’s alpha score of 0.78 [57]. The subscale consists of four questions that measure both physical and verbal victimization that individuals experience from or by peers. The responses to each question ranged from never (scored 0) to 1–2 times (1), 3–4 times (2), 5–6 times (3), and 7 or more times (4). The responses were summed to derived a total score which ranged from 0 to 16.

#### Self-esteem

was assessed with the 10-item Rosenberg’s self-esteem scale. Items are scored on a Likert-like scale with options ranging from “Strongly Disagree” (1 point), “Disagree” (2 points), “Agree” (3 points) to “Strongly Agree” (4 points). The scale has good psychometric properties [58] and has been validated for use among adolescents in Nigeria with a Cronbach’s alpha score of 0.88 [59]. Items 2, 5, 6, 8, 9 were reverse-scored and sum score was derived which ranged from 10 to 40 with higher scores indicating lower self-esteem. The continuous scores were used in analyses.

#### Resilience

was assessed with the 10-item Connor-Davidson resilience scale, which was validated for use in Nigeria with a Cronbach’s alpha score of 0.81 [60]. Each item is rated on a 5-point scale from 0 (‘not true at all’) to 4 (‘true nearly all the time’). The possible total score ranges from 0 to 40 with higher scores indicating higher resilience.

#### Social support

was assessed with the 12-item multidimensional perceived social support scale [61, 62]. The scale has three subscales which inquired about an individual’s perception of the adequacy of support from family, friends, and significant-others’ family. Each subscale comprised four questions. Each item was rated on a 7-point Likert-type response format ranging from 1 - “very strongly agree” to 7- “very strongly disagree.” The possible total score ranged from 12 to 84 with higher total scores corresponding to higher levels of perceived social support, while lower scores indicated perceived unavailability or lack of social support [63]. The scale had been validated for use in Nigeria with a Cronbach’s alpha score of 0.78 [64].

#### Oral hygiene status

Intra-oral examination assessed oral hygiene status using the Simplified Oral Hygiene Index [65]. The oral hygiene score ranges from 0 to 6 categorized into 0.0–1.2 indicating good oral hygiene; 1.3–3.0 as fair oral hygiene; and 3.1–6.0 as poor oral hygiene. The oral hygiene status was dichotomized into good (0.0–3.0) and poor (3.1–6.0) status for the logistic regression analysis.

#### Caries status and complications

Intra-oral examination was also conducted according to the World Health Organization criteria of caries examination to determine the presence of decayed, missing teeth, and filled teeth due to caries using dmft /DMFT indices [66]. Caries status was determined after the oral hygiene status was assessed. Teeth were cleaned with gauze and examined under natural light with dental mirrors without probes. Children were examined seated on a chair. The dmft /DMFT indices were used to categorize the children’s caries status: dmft /DMFT =0 was categorized into caries absent while dmft /DMFT greater or equal to 1 was categorized as caries present. The proportion of children with and without caries was computed.

The dmft /DMFT indices were also used to define the severity of caries for children with caries. Dmft /DMFT greater or equal to 3 was categorized as severe caries while dmft /DMFT scores of 0.1–2.99 was categorized as low caries severity,

Complications associated with carious lesions were assessed with the pufa/PUFA index [67], which was computed for children who had caries. When the pufa/PUFA score was 0, the child was categorized as not having caries complications. Children with a pufa/PUFA score greater than 0 were categorized as having caries complications.

#### Study procedure

Participants were examined seated on a chair in a private area, which was well illuminated with natural light, in the school compound in the presence of a school chaperone. Oral hygiene status was assessed after the questionnaire was filled. The examination was conducted by an examiner and recorded by the assistant. The examiner was calibrated on use of the dmft/DMFT and PUFA/pufa index. The examiner was first calibrated by a consultant and the inter-examiner reliability kappa score was 0.85. Next, an intra-examiner reliability (conducted 1 week after the first examination) was conducted with a kappa score of 0.90.

### Data analysis

The normal distribution of the explanatory variables (ACE, bully victimization, self-esteem, resilience, social support) was determined. The mean (SD) and median (Interquartile range - IOR) of the scores for the explanatory variables were computed. The association between the categorized outcome variables (caries, complications of caries, and poor oral hygiene) and age, sex, socioeconomic status was assessed using chi square test or Mann Whitney U test. The associations with the explanatory variables (ACE, bully victimization, self-esteem, resilience and social support) were determined using the Mann Whitney U test and Kruskal-Wallis test for the variables that were skewed and the t test for those that were normally distributed. Univariate and multivariable logistic regression was conducted to determine the crude and adjusted odds ratios. The models to determine the risk indicators for poor oral hygiene, caries, and complications of caries were adjusted for age, sex and socioeconomic status, which are factors associated with caries, oral hygiene status of children and ACE [68–70]. Statistical significance was conducted with Stata/SE 14.0 for Windows (2015) and measured as *p* < 0.05.

## Results

The data of 1001 (66.6%) of the 1502 collected data with complete information was extracted. Of these, 549 (54.8%) were girls, 962 (96.1%) aged 11–16-year-olds, and 586 (58.5%) children were from middle socio-economic status. Eighty-one (8.1%) children had poor oral hygiene.

Fifty-nine (3.9%) children had caries. The dmft ranged from 0 to 4, with nearly all (99.4%) having 0 dmft. The DMFT ranged from 0 to 6, with most having 0 (94.5%) DMFT. Of the children with caries, 6 (10.2%) had complications. The pufa score was 0 while the PUFA score ranged from 1 to 3. No child with high socioeconomic status had caries complications.

ACE score ranged from 0 to 10 with a median (IQR) score of 1 (3). Also, 679 (67.8%) had one or more ACE. The childhood bully victimization score ranged from 0 to 16 with a median (IQR) score of 1 (5). Also, 619 (62.1%) had been bullied one or more times. The self-esteem score ranged from 3 to 31 with a median (IQR) score of 22 (5). The perceived social support score ranged from 3 to 81 with a with a median (IQR) score of 64 (34). The resilience score ranged from 8 to 50 with a mean (SD) score of 31 (9).

Table 1 highlights the association between age, sex, socioeconomic status and the prevalence and severity of caries, caries complications and oral hygiene status. There was no significant association between sex (*p* = 0.86), socioeconomic status (*p* = 0.36) and age (*p* = 0.72) and the prevalence of caries; nor was there a significant association between sex (*p* = 0.51), socioeconomic status (*p* = 0.19) and age (*p* = 1.00) and caries severity. In addition, there was no significant association between sex (*p* = 0.39), socioeconomic status (*p* = 0.15) and age (p = 1.00) and caries complications; nor was there a significant association between age (*p* = 0.07), and sex (*p* = 0.11) and oral hygiene status. However, there was an association between socioeconomic status and oral hygiene; a greater percentage of children with good oral hygiene than those with fair or poor oral hygiene were from low socioeconomic status (*P* = 0.02).

**Table 1:** Association between sex, socioeconomic status and age and prevalence and severity of caries, caries complication and oral hygiene status in children 6–16 years old resident in Ile-Ife, Nigeria

Variables	Caries *N* = 1001		dmft/DMFT *N* = 59		Caries complications *N* = 59		Oral hygiene *N* = 1001			Total ***N*** = 1001
	Absent ***n*** = 942	Present ***n*** = 59	1–2 ***n*** = 48	**>** 3 ***n*** = 11	Absent ***n*** = 53	Present ***n*** = 6	Good ***n*** = 37	Fair ***n*** = 883	Poor ***n*** = 81	
	Number (%)	Number (%)	Number (%)	Number (%)	Number (%)	Number (%)	Number (%)	Number (%)	Number (%)	Number (%)
**Sex**										
Male	516 (54.8)	33 (55.9)	28 (58.3)	5 (45.5)	31 (58.5)	2 (33.4)	23 (61.2)	490 (55.5)	36 (44.4)	549 (54.8)
Female	426 (45.2)	26 (44.1)	20 (41.7)	6 (54.5)	22 (41.5)	4 (66.6)	14 (38.8)	392 (44.5)	45 (55.6)	452 (145.2)
*p value*	0.86		0.51		0.39		0.11			
**Socioeconomic status**										
Low	272 (28.9)	12 (20.3)	8 (16.7)	4 (36.4)	9 (17.0)	3 (50.0)	19 (51.4)	242 (27.5)	22 (27.2)	284 (28.4)
Middle	547 (58.1)	39 (66.1)	32 (66.7)	7 (63.6)	36 (67.9)	3 (50.0)	16 (43.2)	526 (59.6)	44 (54.3)	586 (58.5)
High	123 (13.0)	8 (13.6)	8 (16.6)	0 (0.0)	8 (15.10)	0 (0.0)	2 (5.4)	114 (12.9)	15 (18.5)	131 (13.1)
*p value*	0.36		0.19		0.15		0.02*			
**Age**										
6–10-year-olds	38 (4.0)	1 (1.7)	1 (2.1)	0 (0.0)	1 (100.0)	0 (0.0)	4 (10.8)	31 (3.5)	4 (4.9)	39 (3.9)
11–16-year-olds	904 (96.0)	58 (58.3)	47 (97.9)	11 (100.0)	52 (89.7)	6 (100.0)	33 (89.2)	851 (96.5)	77 (95.1)	962 (96.1)
*p value*	0.72		1.00		1.00		0.07			

.

Table 2 shows the associations between the explanatory and outcome variables for the study in bivariate analysis. There were no significant differences in ACE score between children with and without caries (*p* = 0.74); with low and high caries severity (*p* = 0.43), with and without caries complications (*p* = 0.60); and with good, fair and poor oral hygiene (*p* = 0.45).

**Table 2:** Adverse childhood experience, bully victimization, self-esteem, resilience and social support scores per prevalence and caries severity, caries complication and oral hygiene status in children 6–16-years-old resident in Ile-Ife, Nigeria

Variables	Caries *N* = 1001			dmft/DMFT *N* = 59			Caries complication *N* = 59			Oral hygiene *N* = 1001			
	Absent *n* = 942	Present *n* = 59	*p*-value	1–2 *n* = 48	**>** 3 *n* = 11	*p*-value	Absent *n* = 53	Present *n* = 6	*p*-value	Good *n* = 37	Fair *n* = 883	Poor *n* = 81	*p*-value
	Median (IQR)	Median (IQR)		Median (IQR)	Median (IQR)		Median (IQR)	Median (IQR)		Median (IQR)	Median (IQR)	Median (IQR)	
ACE	1 (3)	1 (1)	0.74	1 (2)	1 (1)	0.43	1 (1)	1 (0)	0.60	1 (3)	1 (2)	2 (3)	0.45
Bully victimization	1 (5)	2 (5)	0.37	2 (4.5)	4 (7)	0.63	2 (5)	0.5 (2)	0.11	1 (4)	2 (6)	1 (4)	0.17
Self esteem	22 (5)	21 (5)	0.03	21 (5)	20 (5)	0.95	21 (5)	21 (5)	0.97	22 (5)	22 (5)	22 (4)	0.13
Social support	64 (34)	59 (30)	0.08	58.5 (29)	60 (38)	0.63	59 (29)	58.5 (25)	0.65	63 (24)	64 (34)	59.5 (32)	0.50
Resilience^a^	31.7 (8.7)	31 (9.2)	0.55	31.5 (8.1)	32.6 (11.4)	0.70	26 (10)	32.4 (8.4)	0.09	29.3 (9.6)	31.2 (9)	29.7 (9.7)	0.18

^a^Mean (SD) was computed, and the *p* value was computed using t-test

There were no significant associations between childhood bully victimization score and the proportion of children with and without caries (*p* = 0.37), with low and high caries severity (*p* = 0.63), with and without caries complications (*p* = 0.11), and with good, fair and poor oral hygiene (*p* = 0.17).

There were also no significant associations between self-esteem score and the proportion of children with low and high caries severity (*p* = 0.95), with and without caries complications (*p* = 0.97), and with good, fair and poor oral hygiene (*p* = 0.13). There was however, a significant difference in self-esteem score of children with and without caries (*p* = 0.03): the median (IQR) self-esteem score of children with caries was lower than the median (IQR) of children without caries (21(5) vs 22 (5). See Table 2.

There were no significant associations between the social support score and the proportion of children with and without caries (*p* = 0.08), children with low and high caries severity (*p* = 0.63), with and without caries complications (*p* = 0.65); and with good, fair and poor oral hygiene (*p* = 0.50). See Table 2.

There were no significant associations between resilience score and the proportion of children with and without caries (*p* = 0.55), children with low and high caries severity (*p* = 0.70), with and without caries complications (*p* = 0.09); and with good, fair and poor oral hygiene (*p* = 0.18). See Table 2.

Table 3 shows the factors associated with poor oral hygiene after logistic regression analysis. While in the adjusted model, higher self-esteem was associated with better oral hygiene, none of the psychosocial factors had significant association. The higher the self-esteem score, the higher the odds of having poor oral hygiene (AOR: 1.09; 95% CI: 1.01–1.17; *p* = 0.03). In addition, sex and age were associated with poor oral hygiene in the adjusted models. Males had higher odds of having poor oral hygiene than did females (AOR: 1.78; 95% CI: 1.10–2.88; *p* = 0.02). Also, 12–16-year-old children had significantly higher odds of having poor oral hygiene than 6–11-year-old children in the adjusted model (AOR: 0.21; 95% CI: 0.12–0.35; *p* < 0.001).

**Table 3:** Logistic regression analysis to determine psychosocial factors associated with poor oral hygiene in children 6–16-years-old resident in Ile-Ife, Nigeria (*N* = 1001)

Variables	Odds ratio (95% Confidence interval)	***P*** value	Adjusted odds ratio (95% Confidence interval)	***P*** value
**Sex**				
Female	1.00	–	1.00	–
Male	1.58 (1–2.49)	0.05	1.78 (1.1–2.88)	0.02
**Age category**				
6–11 years	1.00	–	1.00	–
12–16 years	0.22 (0.14–0.36)	< 0.001	0.21 (0.12–0.35)	< 0.001
**Socio-economic status**				
Low	1.00	–	1.00	–
Middle	0.83 (0.52–1.31)	0.42	1.04 (0.60–1.81)	0.89
High	1.57 (0.87–2.85)	0.13	1.21 (0.58–2.53)	0.61
**Adverse childhood events**	1.02 (0.90–1.15)	0.75	0.96 (0.83–1.10)	0.55
**Bully victimization**	0.97 (0.91–1.03)	0.29	0.98 (0.91–1.04)	0.47
**Self-esteem**	1.06 (1.00–1.13)	0.06	1.09 (1.01–1.17)	0.03
**Resilience**	0.98 (0.96–1.01)	0.18	1.00 (0.97–1.03)	0.80
**Social support**	0.99 (0.98–1.01)	0.35	1.00 (0.98–1.01)	0.55

Table 4 shows the factors associated with caries after the logistic regression analysis. In the unadjusted model, none of the psychosocial factors was significantly associated with caries. When the model was adjusted, two psychosocial factors became significantly associated with caries: self-esteem and perceived social support. The higher the self-esteem score, the lower was the odds of having caries (AOR: 0.91; 95% CI: 0.85–0.98; *p* = 0.02), and the higher the perceived social support, the lower the odds of having caries (AOR: 0.98; 95% CI: 0.97–1.00; *p* = 0.02).

**Table 4:** Logistic regression analysis to determine psychosocial factors associated with dental caries in children 6–16-years-old resident in Ile-Ife, Nigeria (*N* = 1001)

Variables	Odds ratio (95% Confidence interval)	***P*** value	Adjusted odds ratio (95% Confidence interval)	***P*** value
**Sex**				
Female	1.00	–	1.00	–
Male	0.95 (0.56–1.62)	0.86	0.89 (0.52–1.52)	0.66
**Age category**				
6–11 years	1.00	–	1.00	–
12–16 years	1.95 (0.82–4.6)	0.13	1.94 (0.81–4.67)	0.14
**Socioeconomic status**				
Low	1.00	–	1.00	–
Middle	1.41 (0.81–2.45)	0.23	1.53 (0.78–3.01)	0.21
High	1.04 (0.48–2.25)	0.11	1.67 (0.66–4.23)	0.28
**Adverse childhood events**	0.99 (0.86–1.15)	0.94	0.99 (0.84–1.16)	0.89
**Bully victimization**	1.02 (0.96–1.08)	0.56	1.01 (0.94–1.08)	0.76
**Self-esteem**	0.94 (0.88–1.01)	0.08	0.91 (0.85–0.98)	0.02
**Resilience**	1.01 (0.98–1.04)	0.55	1.00 (0.97–1.03)	0.81
**Social support**	0.99 (0.98–1.00)	0.12	0.98 (0.97–1.00)	0.02

Table 5 shows the factors associated with caries complications. None of the psychosocial factors was associated with caries complications in the unadjusted and unadjusted models. However, the socioeconomic status was significantly associated with caries complications in the adjusted model: The odds of having caries complication was lower for children with middle socio-economic status than for children with low socioeconomic status (AOR: 0.02; 95% CI: 0.00–0.06; *p* = 0.02). No child with high socioeconomic status had caries complications.

**Table 5:** Logistic regression analysis to determine psychosocial factors associated with caries complication in children 6–16-years-old resident in Ile-Ife, Nigeria (*N* = 59)

Variables	Odds ratio (95% confidence interval)	***P*** value	Adjusted odds ratio (95% Confidence interval)	***P*** value
**Sex**				
Female	1.00	–	1.00	–
Male	2.82 (0.47–16.76)	0.26	1.62 (0.08–32.02)	0.75
**Age category**				
6–11 years	1.00	–	1.00	–
12–16 years	0.16 (0.02–1.18)	0.07	0.06 (0.00–1.56)	0.09
**Socioeconomic status**				
Low	1.00	–	1.00	–
Middle	0.47 (0.09–2.59)	0.39	0.02 (0.00–0.60)	0.02
**Adverse childhood events**	0.78 (0.40–1.49)	0.45	0.34 (0.08–1.39)	0.13
**Bully victimization**	0.69 (0.41–1.15)	0.16	0.73 (0.44–1.22)	0.23
**Self-esteem**	1.01 (0.75–1.35)	0.95	1.47 (0.80–2.71)	0.22
**Resilience**	0.91 (0.81–1.02)	0.10	0.99 (0.84–1.18)	0.94
**Social support**	0.99 (0.95–1.03)	0.67	1.01 (0.94–1.09)	0.55

## Discussion

This is the first study conducted in Nigeria that queried if ACE and bully victimization are associated with oral diseases in children and adolescents, and how children and adolescents’ psychological assets and resources are associated with oral diseases. The study findings indicate that though there was no association between presence of caries, caries complication, poor oral hygiene, ACE and bullying, there was an association between presence of caries, self-esteem and social support, and an association between poor oral hygiene and self-esteem. Resilience was not associated with the presence of caries, caries complication and poor oral hygiene. The study was conducted in a community with low caries prevalence confirming previous research [71] and thus, the results may be useful in helping to identify sub-populations with high caries risk who may benefit from strategies to reduce caries risk and ameliorate caries complication [72].

One of the strengths of this study is its large number of participants and the use of instruments that had been validated to measure the various constructs for the study population. In addition, the measure of ACE captures a wide range of possible events. Data was generated through a household survey making the findings representative of the study population. The study also contextualized the interplay between oral health and psychosocial profile in a population of children and adolescents using measures that have not been widely studied.

The study however, is cross-sectional and thus limited in its ability to determine the causality of the associations between psychosocial factors and oral health. The study population was recruited from one of the 774 local government areas of Nigeria thus making the study not representative of all of Nigeria. The sample is also not representative of all children and youths as the participants are in-school with context that differs from that of out-of-school children and adolescents. In addition, although the type of school [73] and body images and size [74] could result in bullying, we did not include this in the data analysis: data on type of school was not available in the primary data. Nonetheless, the study generated several hypotheses that can lead to further research on the inter-relationship between psychosocial factors, culture and oral health.

The study results indicate that the psychosocial factors interact in various ways with oral health problems in children. While the prevalence of caries was lower in children with lower self-esteem and those with higher social support, none of these factors were associated with caries complications. Lower self-esteem was also associated with higher prevalence of poor oral hygiene. All these associations, however, were weak with small effect sizes as indicated by the values of the odds ratios. This is new information on the possible effect of psychosocial factors on oral health though the pathways for the observed association is not clear. Children and adolescents with high perceived social support – in this case, emotional support [75–77] – may have positive outlook on life and may take less risk with their oral health by indulging less with alcohol consumption, tobacco smoking or use of psychoactive substances [78], which reduces the risk for caries [79]. The link between low self-esteem and low prevalence of caries is however, less clear and needs further studies. The association between low self-esteem and poor oral hygiene is plausible but the pathway in this study could not be deciphered –low self-esteem may have impacted negatively on oral hygiene practices, resulting in poor oral hygiene [80, 81], and/or conversely, poor oral hygiene may have resulted in the low self-esteem. Studies on self-esteem and prevalence of caries and poor oral hygiene are needed.

The prevalence of bully victimization was high when compared with reports from other countries. For example, the prevalence of adolescents reporting bully victimization ranged from 14.2–38.9% in Brazil [82–84], to 26% in 40 resource-rich countries [44], 32% in United Kingdom [85], and 47% in Jordan [86]. The prevalence of ACE was also high when compared with the prevalence in other countries - 25% in Hungary [87], 46.3% in the United States of America [88], and 46.4% in the United Kingdom [89]. It was however comparable to the prevalence of 68.2% in Germany [90].

Despite the high prevalence of ACE and bully victimization in this study, they were not significantly associated with caries, caries complications and poor oral hygiene, unlike the associations reported in previous studies [2, 20–23]. There may be several reasons for this. First, it may be too early to detect the impact of ACE and bully victimization on oral health. Adversities in childhood have a dose-response relationship with health problems [91]. In young children and adolescents, the mean number of bully victimization and adverse events is low. Also, adversities in childhood have impact on later-life development [92], so the study may be looking for the impact of ACE too early in the life-time trajectory of events as some define ACE as adverse events that occur in the first 18 years of life [93]. Its impact on the risk for caries and poor oral hygiene may therefore, be less detectable in persons younger than 18 years of age. However, previous studies have investigated and identified the impact of bully victimization on the risk for caries in adolescents younger than 18 years [24, 84]. Studying ACE and bully victimization before age 18 years allows for accurate recollection of events, reduce the risk of recall bias and thereby enables contemporaneous rather than retrospective data collection [94].

Second, stress from ACE and bully victimization may be attenuated by culture. While children who are exposed to stressors from ACE and bully victimization may develop internalizing (anxiety and depression) and externalizing (delinquency and violence against peers) symptoms [95–97], not all children develop these symptoms: these symptoms are moderated by resilience and access to social support [98]. It is also possible that access to social support changes the effects that ACE and bully victimization may have had on oral health. Also, the collectivism culture of the ethnic group of the study population – the Yorubas [99] – may moderate the perception of stress from ACE and bully victimization. Ngo and Le [98] reported that collectivism dampened the impact of some stressors by enabling individuals to marshal and rely on support and resources from others. Social support, which is usually highly available in collective societies, may ameliorate the impact of negative life events. However, when the individual’s self-esteem is affected negatively by these life events, the negative impact of ACE and bully victimization on the risk of caries may be stronger.

Third, the relatively high prevalence of ACE and bully victimization observed in the study population might have imparted a norm status to them. If these phenomena are common, there is less variation among participants in the chances of experiencing them and consequently they cannot explain differences in oral health outcomes [100]. This complex inter-relationship between psychosocial factors and oral diseases needs further study.

## Conclusion

The relationship between psychosocial factors and oral diseases is complex: While self-esteem and perceived social support had a protective effect on the risk of caries, they were not protective against poor oral hygiene. In all cases, the associations indicated weak effects. ACE and bully victimization were not associated with caries, caries complications, or poor oral hygiene. The possible effect of culture in moderating these relationships needs to be explored.

## Data Availability

This is a secondary analysis of a primary data we accessed from the PI with the primary data. The data used for this study is presented in the manuscript. The PI will the primary data is willing to make the data available on request.

## Abbreviations

ACE: Adverse childhood experiences
AOR: Adjusted odds ratio
CI: Confidence interval
dmft: Decay missing filled tooth
DMFT: Decay Missing Filled tooth
IQR: Interquartile range
OR: Odds ratio
